# A Multivariate Poisson Deep Learning Model for Genomic Prediction of Count Data

**DOI:** 10.1534/g3.120.401631

**Published:** 2020-09-15

**Authors:** Osval Antonio Montesinos-López, José Cricelio Montesinos-López, Pawan Singh, Nerida Lozano-Ramirez, Alberto Barrón-López, Abelardo Montesinos-López, José Crossa

**Affiliations:** * Facultad de Telemática, Universidad de Colima, Colima, 28040, México; † Departamento de Estadística, Centro de Investigación en Matemáticas (CIMAT), Guanajuato, 36023, México; ‡ Biometrics and Statistics Unit, Genetic Resources Program, International Maize and Wheat Improvement Center (CIMMYT), Km 45 Carretera Mexico-Veracruz, CP 52640, Mexico; § Department of Animal Production (DPA), Universidad Nacional Agraria La Molina, Av. La Molina s/n La Molina, 15024, Lima, Perú; ** Departamento de Matemáticas, Centro Universitario de Ciencias Exactas e Ingenierías (CUCEI), Universidad de Guadalajara, 44430, Jalisco, México; †† Colegio de Post-Graduados, Montecillos Texcoco. Edo. de Mexico

**Keywords:** Genomic selection and genomic prediction, count data of wheat lines, multivariate Poisson deep neural network, univariate Poisson deep neural network, Poisson regression models, GenPred, Shared data resources

## Abstract

The paradigm called genomic selection (GS) is a revolutionary way of developing new plants and animals. This is a predictive methodology, since it uses learning methods to perform its task. Unfortunately, there is no universal model that can be used for all types of predictions; for this reason, specific methodologies are required for each type of output (response variables). Since there is a lack of efficient methodologies for multivariate count data outcomes, in this paper, a multivariate Poisson deep neural network (MPDN) model is proposed for the genomic prediction of various count outcomes simultaneously. The MPDN model uses the minus log-likelihood of a Poisson distribution as a loss function, in hidden layers for capturing nonlinear patterns using the rectified linear unit (RELU) activation function and, in the output layer, the exponential activation function was used for producing outputs on the same scale of counts. The proposed MPDN model was compared to conventional generalized Poisson regression models and univariate Poisson deep learning models in two experimental data sets of count data. We found that the proposed MPDL outperformed univariate Poisson deep neural network models, but did not outperform, in terms of prediction, the univariate generalized Poisson regression models. All deep learning models were implemented in Tensorflow as back-end and Keras as front-end, which allows implementing these models on moderate and large data sets, which is a significant advantage over previous GS models for multivariate count data.

The selection of the best candidate genotypes is done by observing the phenotype of traits of interest in conventional breeding programs, which is very expensive since all the candidate genotypes have to be planted in the field. For these reasons, phenotypic selection (PS) is being replaced by genomic selection (GS) in many crops around the world, since it makes it possible to select the best candidate genotypes early in time using a statistical learning model that is able to learn the relationship between the genotyped (marker data) and phenotyped information of the training set (Meuwissen *et al.*, 2001). GS is a technology that is transforming the conventional breeding process since we select the genotypes of interest early in time and we need only the genotypic information for the genotypes we want to select. However, since there is no universal statistical machine learning model that always performs the best for all types of data, we need specific algorithms for some types of data (Wolpert and Macready 1997). For example, it is very well documented that for each type of response variable there are specific predictive machines that are more appropriate for each circumstance. For example, multiple regression performs well for continuous data with linear patterns in the data, while logistic regression is a good option for binary data with linear patterns, and multinomial regression is a reasonable option for categorical response variable with linear patterns. However, there is evidence that deep learning (DL) models are good candidates for non-linear patterns in the data (Chollet and Allaire 2017; Patterson and Gibson 2017). But to implement DL models we also need to be careful since it is very well documented that for continuous outcomes, the mean square error (mse) loss function is one of the best options, although it is not a reasonable option for binary and categorical outcomes (Chollet and Allaire 2017; Patterson and Gibson 2017). For binary and categorical response variables, the best options are the binary-cross-entropy and categorical-cross-entropy loss functions, respectively (Chollet and Allaire 2017; Patterson and Gibson 2017). These examples illustrate that unfortunately there is no universal statistical machine learning model that works well for all types of data (Wolpert and Macready 1997).

In addition to the appropriate selection of the statistical machine learning model, there are other issues that need to be considered to successfully implement GS. Some of these issues are: (a) to select a representative (training) set (Guo *et al.*, 2019), (b) to guarantee the quality of genotypic and phenotypic data in the training set (Edwards *et al.*, 2019), and (c) to have a representative sample (good coverage) of the markers in the complete genome. The empirical evidence of the usefulness of GS continues to grow, showing that it is a power tool that can revolutionize the way plant breeders perform the selection of candidate genotypes (Crossa *et al.*, 2013; Meuwissen *et al.*, 2013; Crossa, *et al.*, 2017; Vivek *et al.*, 2017; Smallwood *et al.*, 2019; Môro *et al.*, 2019; Salam and Smith 2016). Some research studies showing that the GS methodology works as well as PS are given next: Vivek *et al.* (2017), Smallwood *et al.* (2019), Môro *et al.* (2019), and Salam and Smith (2016). However, it is important to point out that the empirical evidence supports that GS is not better than PS, since no relevant differences are observed between GS and PS, but GS has the advantage over PS because it requires fewer resources, reduces the cost per cycle and shortens the generation interval (Crossa *et al.*, 2017; Farah *et al.*, 2016). GS could become a key selection methodology in plant science, since more high-quality data are becoming available, and predictive algorithms will be able to combine different types of data more efficiently and thereby improve the prediction accuracy. Although GS is not yet the main tool for plant breeders, it has been implemented in many crops like maize, wheat, chickpea, cassava and rice, among others (Crossa *et al.*, 2017; Roorkiwal *et al.*, 2016; Wolfe *et al.*, 2017; Huang *et al.*, 2019), and the number of breeding programs that are moving from conventional breeding to GS continues to grow. As mentioned above, one important aspect of the successful implementation of GS is correctly selecting the algorithm for prediction. However, for multivariate count data, only univariate models are available, such as the Poisson deep neural network (PDNN) model proposed by Montesinos-López, unpublished results), which can be implemented very efficiently for moderate [at least ten of thousands of observations (rows)] or large data sets (more than twenty-five thousand observations; Emmert-Streib *et al.*, 2020).

Count data are common in many domains since they reflect the number of occurrences of an outcome variable measured in a fixed period of time (for example, per hour or day), area (for example, per square meter) or volume (for example, per cubic meter). Some examples in particular domains are in information technology (number of visits per day to a web site; number of spam emails received per day), demography (number of families in poverty in a city or region; number of accidents per day in a city), animal science (number of sick animals per herd; number of offspring per sow), social science (number of religious families per region or area), chemistry (number of red blood cells per millimeter), physics (number of alpha particles emitted from a source in a given time interval), etcetera. Count data are also common in plant breeding since they allow, for example, measuring the number of panicles per plant, number of seeds per plant, number of infected spikelets per plant, days to heading, days to maturity, and days to germination, among others (Montesinos-López *et al.*, 2016; Montesinos-López *et al.*, 2017). Count data take on values of 0, 1, 2,... with an unrestricted upper limit. Usually, count data are analyzed incorrectly with ordinal least square regression or models for continuous outcomes, even though there is a lot of evidence that the Poisson or negative binomial family of regressions are better alternatives for modeling count data. The Poisson family has the inconvenience that it assumes that the variance is equal to the mean, that is, many times it is unable to capture over-dispersion efficiently, but the negative binomial family allows modeling this problem of over-dispersion appropriately most of the time.

For multivariate data under Poisson distribution, it is not possible to implement closed form Bayesian estimation; only approximate Bayesian estimation is available, but it is inefficient (Montesinos-López *et al.*, 2015, 2016, and 2017) since there is no analytical Gibbs sampler available to draw samples of the posterior distribution of the parameters of interest. However, these observations are also valid for classic estimations of multivariate Poisson and negative binomial distribution. For this reason, Montesinos-López, unpublished results) proposed the univariate PDNN model for count data using Tensorflow as back-end and Keras in R as front-end (Chollet and Allaire 2017); this framework is useful for large data sets. Deep learning (DL) models are generalized artificial neural networks, but with more than one hidden layer. Hidden layers consist of non-linear transformations applied to the input information with the goal of filtering the data and removing the noise in a way that helps to increase the prediction performance in the testing set. For complex input data, usually more hidden layers are required to improve the prediction performance. DL models try to mimic the functioning of our brain when performing complex tasks.

Successful applications of DL models are applied for tasks like: face recognition, voice recognition (Chollet and Allaire 2017), self-driving cars that are capable of sensing their environment and moving safely with little or no human input (Chollet and Allaire 2017), cancer and skin predictions using images as information (Kadampur and Al Riyaee 2020), human resource selection in companies, genomic selection (Montesinos-López *et al.*, 2018a, b; Montesinos-López *et al.*, 2019a, b), etcetera. Empirical evidence shows that DL models are competitive (with at least the same performance) with conventional statistical machine learning models, mostly for larger data sets. However, until now there is no flexible framework that allows researchers without a strong background in computer science and statistics to implement univariate and multivariate models for modeling multivariate count data in DL; for this reason, we propose a deep neural network framework for implementing multivariate count models. This framework captures non-linearity in a better way than conventional statistical machine learning models since it is able to use many hidden layers that apply no linear transformations. The proposed multivariate Poisson deep neural network (MPDN) model for count data uses the negative log-likelihood of a Poisson distribution as the loss function and the exponential activation function for each trait in the output layer, to ensure that all predictions are positive.

## Material And Methods

### Univariate generalized Poisson regression model

The Poisson distribution with parameter μ belongs to the exponential family and its probability function is equal to:

$$f\left(y_{i}\right)=\frac{e^{-\mu_{i}}\mu_{i}^{y_{i}}}{y_{i}!},\;\;y_{i}=0,\;1,\;2,\cdots ,$$

where $y_{i}$=0,1,2,3,… is the value of the counting variable associated with unit i, given a set of explanatory variables. The mean and variance of a Poisson random variable are equal to $E\left(y_{i}\right)=Var\left(y_{i}\right)=\mu_{i}$. This Poisson distribution is often used to model responses that are “counts.” Given that our training set is composed of pairs of inputs ($y_{i}$, $x_{i}^{T}$) with $x_{i}^{T}=[x_{i1},\;\ldots ,x_{ip}]$, for i=1, 2,…,n, the logarithm of the likelihood is given by:

$$logf\left(y_{i}\right)=log\left(\overset{n}{\underset{i=1}{\prod }}\frac{e^{-\mu_{i}}\mu_{i}^{y_{i}}}{y_{i}!}\right)=\overset{n}{\underset{i=1}{\sum }}[-\mu_{i}+y_{i}log\left(\mu_{i}\right)-log\left(y_{i}!\right)].$$

According to Stroup (2012), the specification of a generalized Poisson regression model is given as:

Predictor: $log\left(\mu_{i}\right)=\eta +\overset{p}{\underset{j=1}{\sum }}x_{ij}\beta_{j}$

Distribution: $y_{i}∼Poisson\left(\mu_{i}\right)$

Link function: log

where η is the intercept, $x_{ij}$ is the jth independent variable measured in observation i, where j=1,2,..,p, $\beta_{j}$ is the beta coefficient corresponding to the independent variable j. Thus, the expected value is $E\left(y_{i}\text{|}x_{i}^{T}\right)=\mu_{i}=\text{exp}(\eta +\overset{p}{\underset{j=1}{\sum }}x_{ij}\beta_{j})$. Since the link function is the log function, this means that the *inverse link function* is the exponential function, which is called the *activation function* in the specification of the multivariate Poisson deep neural network model. The optimization process can be performed by minimizing the negative loglikelihood (called loss function=LL). However, when the number of independent variables (p) is larger than the number of observations, it is better to use a penalized version of the negative loglikelihood (LL), which is equal to:

$$LL=-\overset{n}{\underset{i=1}{\sum }}[-\mu_{i}+y_{i}log\left(\mu_{i}\right)]+\lambda \left(\left(1-\alpha \right)[\overset{p}{\underset{j=1}{\sum }}\beta_{j}^{2}+\alpha \overset{p}{\underset{j=1}{\sum }}\left|\beta_{j}\right|]\right)$$

where λ is the tuning hyper-parameter that can be chosen by cross-validation and α is a parameter that causes Ridge penalization, Lasso penalization or a mixture of both. For example, when α=0, the LL corresponds to a univariate Generalized Poisson Ridge Regression (GPRR); when α=1, the LL corresponds to a univariate Generalized Poisson Lasso Regression (GPLR), and when 0<α<1, the LL corresponds to a univariate Generalized Poisson elastic net regression (GPER). The optimization of this loss function (LL) was done using the R package glmnent (Lasso and Elastic-Net Regularized Generalized Linear Models) (Friedman *et al.*, 2010). The selection of the tuning hyper-parameter (λ) was performed with 10 fold cross-validations created with each training set.

### Multi-trait Poisson deep neural network (MPDN) model

**Figure 1:**
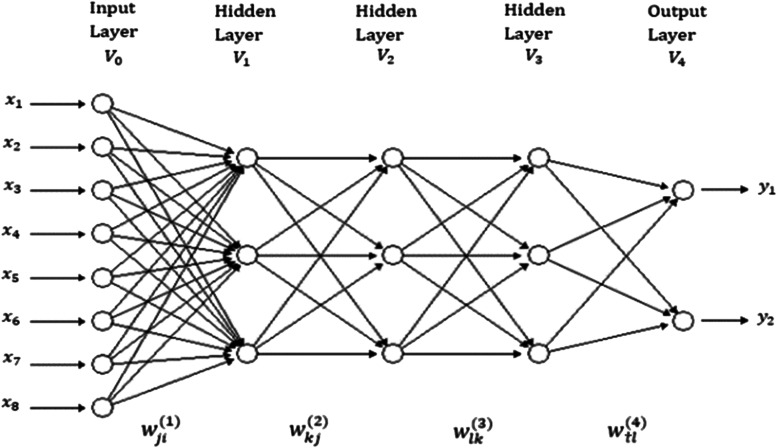
A feedforward deep neural network with one input layer, three hidden layers and one output layer. There are eight neurons in the input layer that correspond to the input information, three neurons in each of three hidden layers, with two neurons in the output layers that correspond to the traits that will be predicted.

The topology of the multi-trait Poisson deep neural network (MPDN) consists of a feedforward neural network with an input layer (8 inputs, as shown in Figure 1), at least one hidden layer (3, as shown in Figure 1) and an output layer (with at least two outputs). The input layer receives all the independent variables that are supposed to be related to the output (in our case, environments and lines taking into account the marker data). Each neuron of the first hidden layer receives as input a net input that is a weighted sum of those independent variables with their corresponding weights, plus an intercept ($\overset{p}{\underset{i=1}{\sum }}w_{ji}^{\left(1\right)}x_{i}+b^{\left(j\right)}\;\text{for}\;j=1,\ldots ,\;M_{1})$ to which a nonlinear transformation is applied to capture complex patterns (in our case, for all neurons in all hidden layers we applied the ReLU nonlinear transformation, also called activation function). Then the output of the neurons of the first hidden layers ($V_{1j}=g_{1}\left(\overset{p}{\underset{i=1}{\sum }}w_{ji}^{\left(1\right)}x_{i}+b^{\left(j\right)}\right)$ for $j=1,\ldots ,\;M_{1}$) were used as input for the neurons of the second hidden layer, and again a net input was created from the output of the neurons of the first hidden layers ($\overset{M_{1}}{\underset{j=1}{\sum }}w_{kj}^{\left(2\right)}V_{1j}+b^{\left(k\right)}\;\text{for}\;k=1,\ldots ,\;M_{2}$), which was transformed with a nonlinear transformation, $g_{2}$ (also ReLU) to produce the output of each neuron. Then the output of each of the neurons in the second hidden layer ($V_{2k}=g_{2}\left(\overset{M_{1}}{\underset{j=1}{\sum }}w_{kj}^{\left(2\right)}V_{1j}+b^{\left(k\right)}\right)$ for $k=1,\ldots ,\;M_{2}$) was used as input for the neurons of the third hidden layer, for which its corresponding net input was equal to ($\overset{M_{2}}{\underset{k=1}{\sum }}w_{lk}^{\left(3\right)}V_{2k}+b^{\left(l\right)}$ for $l=1,\ldots ,\;M_{3})$, after applying the nonlinear transformation (also ReLU) produced as output of each neuron ($V_{3l}=g_{3}\left(\overset{M_{2}}{\underset{k=1}{\sum }}w_{lk}^{\left(3\right)}V_{2k}+b^{\left(l\right)}\right)$ for $l=1,\ldots ,\;M_{3}$). Since we are assuming only three hidden layers, finally the net input of each of the outputs is created with the output of all neurons in the third hidden layer ($\overset{M_{3}}{\underset{l=1}{\sum }}w_{tl}^{\left(4\right)}V_{3l}+b^{\left(t\right)}$ for t=1,…T) to which we apply an exponential activation function (transformation) for each output to guarantee positive outcomes ($y_{t}=exp\left(\overset{M_{3}}{\underset{l=1}{\sum }}w_{tl}^{\left(4\right)}V_{3l}+b^{\left(t\right)}\right)$ for t=1,…T) on the same scale of the count data.

All model equations for a MPDN with p inputs, $M_{1}$ hidden neurons (units) in hidden layer 1, $M_{2}$ hidden units in hidden layer 2, $M_{3}$ hidden units in hidden layer 3, and T outputs, are given by the following equations (1-4):

$$V_{1j}=g_{1}\left(\overset{p}{\underset{i=1}{\sum }}w_{ji}^{\left(1\right)}x_{i}+b^{\left(j\right)}\right)\;\;\text{for}\;\;j=1,\ldots ,\;M_{1}$$ (1)

$$V_{2k}=g_{2}\left(\overset{M_{1}}{\underset{j=1}{\sum }}w_{kj}^{\left(2\right)}V_{1j}+b^{\left(k\right)}\right)\;\;\text{for}\;\;k=1,\ldots ,\;M_{2}$$ (2)

$$V_{3l}=g_{3}\left(\overset{M_{2}}{\underset{k=1}{\sum }}w_{lk}^{\left(3\right)}V_{2k}+b^{\left(l\right)}\right)\;\text{for}\;l=1,\ldots ,\;M_{3}$$ (3)

$$y_{t}=exp\left(\overset{M_{3}}{\underset{l=1}{\sum }}w_{tl}^{\left(4\right)}V_{3l}+b^{\left(t\right)}\right)\;\;\text{for}\;\;t=1,\ldots T$$ (4)

where equation (1) produces the output of each of the neurons in the first hidden layer, equation (2) produces the output of each of the neurons in the second hidden layer, equation (3) produces the output of each of the neurons in the third hidden layer and finally, equation (4) produces the output of the T count response variables. The learning process is achieved with the weights ($w_{ji}^{\left(1\right)},w_{kj}^{\left(2\right)},w_{lk}^{\left(3\right)}\;$ and $w_{tl}^{\left(4\right)})$ and biases ($b^{\left(j\right)},b^{\left(k\right)},b^{\left(l\right)}\;$ and $b^{\left(t\right)})$ that correspond to the first hidden layer, second hidden layer, third hidden layer and the output layer, respectively. To obtain the outputs of each of the neurons in the three hidden layers ($g_{1},\;g_{2}\;\text{and}\;g_{3}),\;$we used the rectified linear activation unit (ReLU) activation function. However, for the output layer, we used the exponential activation functions ($g_{4})$ [see equation (4)], since the response variables we wanted to predict are counts. However, the ReLU activation function can also be used for count data because it guarantees positive predicted values (Chollet and Allaire 2017; Patterson and Gibson 2017). In theory, a neural network with enough hidden units can approximate any arbitrary functional relationships (Cybenko 1989; Hornik 1991). The proposed MPDN model was implemented in the Keras library as front-end and in Tensorflow as back-end (Chollet and Allaire 2017). It is important to point out that when the MPDN model (described in equations 1-4) has only one output (only one response variable), this model is reduced to the univariate Poisson deep neural (UPDN) model.

To select the hyper-parameters, we used a different grid search for each data set. For data set 1, the grid search method contained 10 combinations of hyper-parameters with five neurons (120, 160, 200, 240, 280) and two lambda parameters for the Lasso penalization (0.001, 0.01); the remaining hyper-parameters were fixed (batch_size = 273, learning_rate = 0.001, 0% of dropout, ReLU activation function for hidden layers, Poisson loss function, validation split was 20% of the outer training set, number of epochs used was 1000 in the outer training set, and an adam optimizer). By epoch we mean the number of times the learning algorithm will work across the entire training data set. For data set 2, we used 16 combinations of hyper-parameters: four values of neurons (400, 600, 800, 1000), two values of percent dropout (0, 0.05), and two values of the lambda parameter for Lasso penalization (0.001, 0.01). The remaining hyper-parameters were fixed as for data set 1, except for the batch size, which now was set at 500. It is important to point out that each of the 10 (data set 1) and 16 (data set 2) combinations were run with these fixed hyper-parameters under 1, 2, 3 and 4 hidden layers, using the early stopping approach that allows selecting the optimal number of epochs. From combinations 10 and 16, we selected the best combination for each hidden layer (1, 2, 3 and 4) in terms of the lower mean square error of prediction inside each outer training set since this metric is one of the default metrics in Keras. Then, with this optimal combination of hyper-parameters, the model was refitted using all the information of the outer training set. Finally, predictions were made for the corresponding outer testing set using the estimated model with the refitted model. This process was done in each of the five folds. The optimal hyper-parameters for each hidden layer (1, 2, 3, 4) were selected using the grids given above; therefore, all these models belong to the MPDN and UPDN (same as the MPDN, except that it only contains one output) even though when only one hidden layer is used, these models are only a conventional artificial neural network.

Furthermore, the generalized Poisson regression model depends on the value of alpha (α), and with different alphas we get a different model. For this reason, 5 models were built using different values of α. Table 1 shows the 13 models generated, 4 belonging to the MPDN, 4 to the UPDN and 5 to generalized Poisson regression.

**Table 1:** Proposed and implemented models. NN denotes that the parameters are not needed for a model. Alpha is the parameter α and is needed only in generalized Poisson regression models. UPDN_1 denotes the univariate Poisson deep neural network with 1 hidden layer, UPDN_2 denotes a UPDN with two hidden layers and so on. MPDN_1 denotes the multivariate Poisson deep neural network with 1hidden layer, MPDN_2 denotes a MPDN with two hidden layers, and so on

Model	Model name	Abbreviation of model	Hidden layer	Alpha
1	Univariate Poisson deep neural network	UPDN_1	1	NN
2	Univariate Poisson deep neural network	UPDN_2	2	NN
3	Univariate Poisson deep neural network	UPDN_3	3	NN
4	Univariate Poisson deep neural network	UPDN_4	4	NN
5	Multivariate Poisson deep neural network	MPDN_1	1	NN
6	Multivariate Poisson deep neural network	MPDN_2	2	NN
7	Multivariate Poisson deep neural network	MPDN_3	3	NN
8	Multivariate Poisson deep neural network	MPDN_4	4	NN
9	Univariate Generalized Poisson Elastic net regression	GPR_0.75	0	0.75
10	Univariate Generalized Poisson Elastic net regression	GPR_0.5	0	0.5
11	Univariate Generalized Poisson Elastic net regression	GPR_0.25	0	0.25
12	Univariate Generalized Poisson Lasso regression	GPR_Lasso	0	1
13	Univariate Generalized Poisson Ridge regression	GPR_Ridge	0	0

### Data

#### Phenotypic data set 1:

The phenotypic data set used included 182 spring wheat lines developed by the International Maize and Wheat Improvement Center (CIMMYT) that were assembled and evaluated for resistance to *Fusarium graminearum* in three experiments conducted at El Batan experiment station in Mexico in 2011. For the application, we call these three experiments Env1, Env2, and Env3. In all the experiments (environments), the genotypes were arranged in a randomized complete block design, in which each plot comprised two 1-m double rows separated by a 0.25-m space. *Fusarium* head blight (FHB) severity data were collected 20 and 30 days (d) before maturity by counting symptomatic spikelets on five randomly selected spikes in each plot. We used the counts collected at 20 d as trait 1 and the counts collected at 30 d as trait 2.

#### Genotypic data set 1:

DNA samples were extracted from young leaves 2–3 weeks old, taken from each line, using Wizard Genomic DNA purification (Promega) and following the manufacturer’s protocol. DNA samples were genotyped using an Illumina 9K SNP chip with 8632 single nucleotide polymorphisms (SNPs) (Cavanagh *et al.*, 2013). For a given marker, the genotype for the ith line was coded as the number of copies of a designated marker-specific allele carried by the ith line (absence = zero and presence = one). SNP markers with unexpected AB (heterozygous) genotype were recoded as either AA or BB, based on the graphical interface visualization tool of GenomeStudio (Illumina) software. SNP markers that did not show clear clustering patterns were excluded. In addition, 66 simple sequence repeat markers were screened. After filtering the markers for 0.05 minor allele frequency and deleting markers with 0.10% of no calls, the final set of SNPs included 1635 SNPs.

#### 1. Phenotypic data set 2:

This data set contains 438 lines for which three diseases were recorded. *Pyrenophora tritici-repentis* (PTR) that causes a disease originally named yellow spot but also known as tan spot, yellow leaf spot, yellow leaf blotch or helminthosporiosis. *Parastagonospora nodorum* (SN) is a major fungal pathogen of wheat fungal taxon that includes several plant pathogens affecting the leaves and other parts of the plants.

*Bipolaris sorokiniana* (SB) is of economic importance as the cause of seedling diseases, common root rot and spot blotch of several crops like barley and wheat. The 438 wheat lines were evaluated in the greenhouse for several replicates during a long period of time. The replicates were considered as different environments (Env1, Env2, Env3, Env4, Env5, and Env6). The total number of observations were 438×6=2628 observations for which the three traits were measured.

#### Genotypic data set 2:

DNA samples were extracted from each line, following the manufacturer’s protocol. DNA samples were genotyped using 67,436 single nucleotide polymorphisms (SNPs). For a given marker, the genotype for the ith line was coded as the number of copies of a designated marker-specific allele carried by the ith line (absence = zero and presence = one). SNP markers with unexpected heterozygous genotype were recoded as either AA or BB. We kept those markers that had less than 15% missing values. After that, we imputed the markers using observed allelic frequencies. We also removed markers with MAF < 0.05. After Quality Control and imputation, a total of 11,617 SNPs were still available for analysis.

### Metrics used to measure prediction performance

**Figure 2:**
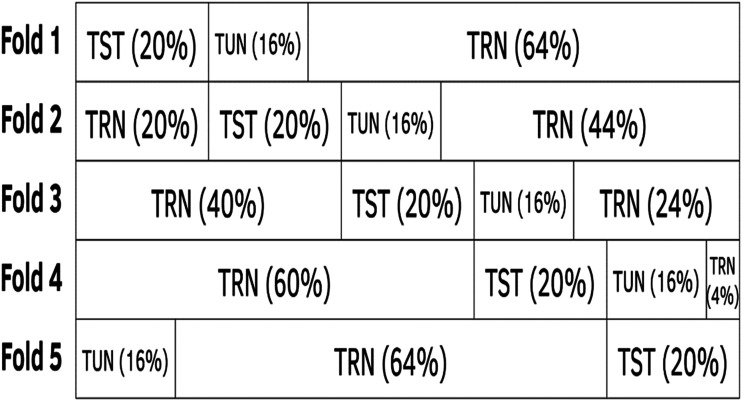
Strategy of fivefold cross-validation. In each fold, 20% of the data were used for testing (TST), 64% for training (TRN) and 16% for the tuning process (TUN). This strategy was used only for deep learning (MPDN and UPDN) models.

Cross-validation is a strategy for model selection and is also used to evaluate the prediction performance in unseen data. For this reason, we used cross-validation to evaluate the prediction performance in unseen data. Since our data contain the same lines in I environments, we used a type of cross-validation that mimics a situation where lines were evaluated in some environments for all traits but where some lines were missing in other environments. We implemented a fivefold cross-validation, where four folds were used for training and one fold for testing. We reported the average prediction performance for the test data in terms of mean square error of prediction (MSE), mean arctangent absolute percentage error (MAAPE) for each environment and average Pearson correlation (APC) for each environment. It is important to point out that the process for tuning the hyper-parameter (λ) in the generalized Poisson regression (GPR_0.75, GPR_0.5, GPR_0.25, GPR_Lasso and GPR_Ridge) was done with ten-fold cross-validation, while the tuning process for the Poisson deep neural network models (MPDN and UPDN) was done in each of the five folds of the fivefold cross-validation (see Figure 2) strategy; in each fold, 20% of the data were used for testing (TST), 64% of the information for training (TRN) and 16% for tuning (TUN) (see Figure 2). Each of the 10 (data_set_1) and 16 (data_set_2) combinations of the grid search was trained with the training set in each fold and its prediction performance was evaluated in the tuning (TUN) set. After selecting in terms of MSE the best combination of hyper-parameters, the model was refitted but using the whole training set (80% of data, since the TRN+TUN sets were joined) in each fold. Finally, for each testing set, we computed each of the three metrics (MSE, MAAPE and APC) with its corresponding standard error (SE) which were computed using 500 bootstrap samples (of observed and predicted values of the testing set); then the average of the 5 folds and its SE was reported as a measure of prediction performance and variability in each metric. It is important to point out that the five fold cross-validation strategy was implemented with only 1 replication.

### Data availability

The phenotypic and genotypic data used in this study are contained in the following R files Data_set 1.RData and Data_set 2.RData, available at the following link: http://hdl.handle.net/11529/10548438.

## Results

The results are given in three sections. The first section provides the distribution of the phenotypic information of both data sets. The second provides the results of the 13 models for data_set_1 and data_set_2.

### Distribution of the phenotypic data sets

**Figure 3:**
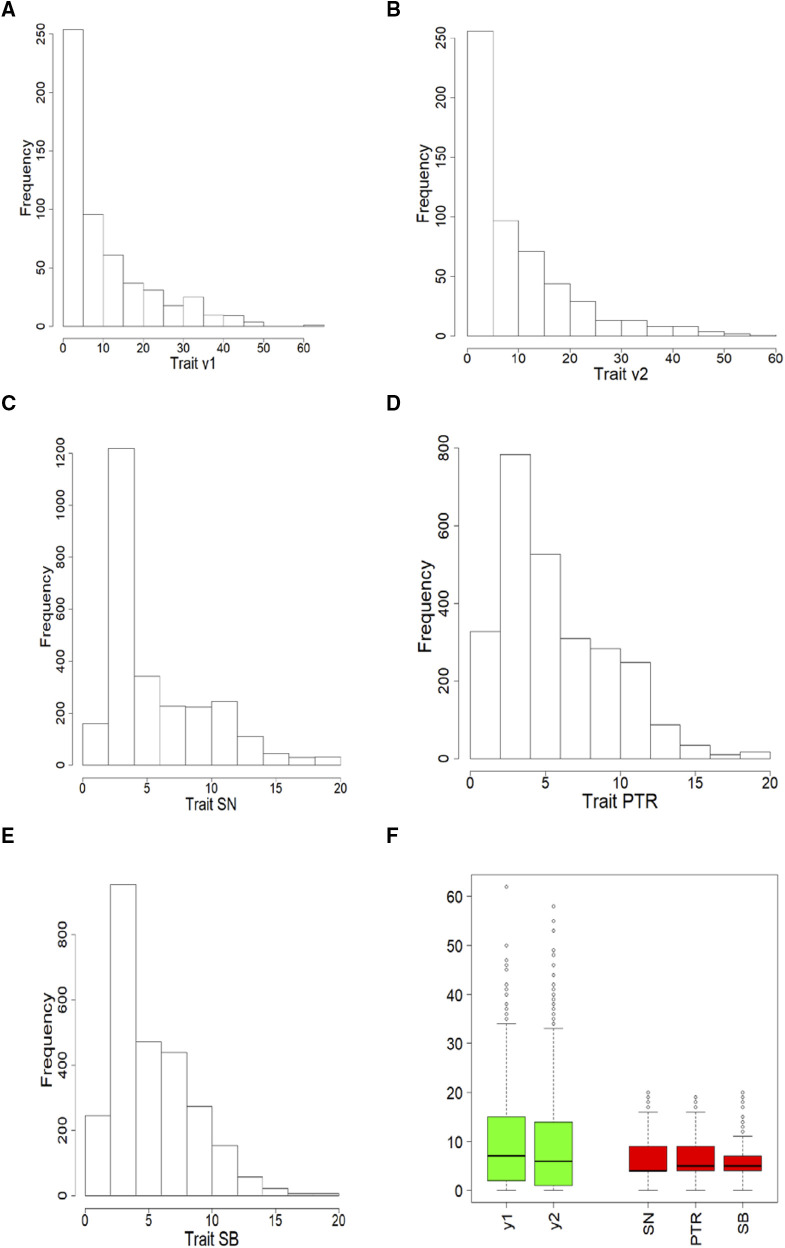
Histograms of phenotypic data of data set 1 [traits y1 (A) and y2 (B)] and of data set 2 [traits SN (C), PTR (D), and SB (E)]. In (F) is the boxplot of the five traits of both data sets.

Table 2 shows the distribution of each of the two count traits (y1, y2) of data set 1 and the three count traits (SN, TPR and SB) of data set 2. Figure 2A and Figure 2B indicate that for the two traits of data set 1, the closer to zero the counts are, the larger the frequency; this is clearly an asymmetric distribution and the counts are between zero and less than 70. For the three traits (Figure 2C, 2D and 2E) of data set 2, the distribution of the three traits is also asymmetric, but now the counts with larger frequencies are above zero and under 5, and the counts are between zero and 20. Figure 2F gives a box plot of the five traits that shows the minimum, maximum, mean, median, quantile 25% and quantile 75% for each trait (Figure 3).

**Table 2:** Prediction performance of data set 1 in terms of mean square error (MSE), mean arctangent absolute percentage error (MAAPE) and Average Pearson Correlation (APC) without taking into consideration genotype×environment interaction (WI) and taking it into account (I) in the 13 models. SE_1 denotes the standard error of the MSE, SE_2 denotes the standard error of the MAAPE and SE_3 denotes the standard error of the APC

Model	Interaction	Trait	MSE	SE_1	MAAPE	SE_2	APC	SE_3
UPDN_1	WI	y1	49.792	7.579	0.596	0.024	0.546	0.054
UPDN_1	WI	y2	51.319	7.742	0.662	0.015	0.523	0.06
		Average	50.556	7.661	0.629	0.020	0.535	0.057
UPDN_2	WI	y1	46.03	4.51	0.607	0.023	0.55	0.043
UPDN_2	WI	y2	46.611	7.902	0.668	0.024	0.535	0.067
		Average	46.321	6.206	0.638	0.024	0.543	0.055
UPDN_3	WI	y1	46.08	4.969	0.623	0.021	0.553	0.048
UPDN_3	WI	y2	48.441	6.686	0.681	0.021	0.524	0.061
		Average	47.261	5.828	0.652	0.021	0.539	0.055
UPDN_4	WI	y1	44.609	5.645	0.625	0.018	0.554	0.032
UPDN_4	WI	y2	49.637	7.102	0.673	0.015	0.523	0.078
		Average	47.123	6.374	0.649	0.017	0.539	0.055
MPDN_1	WI	y1	45.507	10.464	0.581	0.047	0.87	0.025
MPDN_1	WI	y2	37.971	9.21	0.583	0.048	0.865	0.026
		Average	41.739	9.837	0.582	0.048	0.868	0.026
MPDN_2	WI	y1	45.294	10.069	0.591	0.048	0.863	0.026
MPDN_2	WI	y2	36.453	9.294	0.582	0.049	0.86	0.027
		Average	40.874	9.682	0.587	0.049	0.862	0.027
MPDN_3	WI	y1	48.682	10.454	0.595	0.048	0.854	0.027
MPDN_3	WI	y2	39.857	8.977	0.612	0.047	0.853	0.028
		Average	44.270	9.716	0.604	0.048	0.854	0.028
MPDN_4	WI	y1	49.999	10.283	0.625	0.046	0.831	0.031
MPDN_4	WI	y2	41.182	8.96	0.614	0.048	0.834	0.031
		Average	45.591	9.622	0.620	0.047	0.833	0.031
GPR_L1_0.5	WI	y1	39.964	8.811	0.583	0.047	0.889	0.022
GPR_L1_0.5	WI	y2	36.945	9.926	0.592	0.048	0.861	0.025
		Average	38.455	9.369	0.588	0.048	0.875	0.024
GPR_L1_0.25	WI	y1	36.732	7.982	0.577	0.047	0.896	0.021
GPR_L1_0.25	WI	y2	35.138	9.154	0.592	0.049	0.867	0.025
		Average	35.935	8.568	0.585	0.048	0.882	0.023
GPR_L1_0.75	WI	y1	41.012	9.051	0.583	0.047	0.886	0.022
GPR_L1_0.75	WI	y2	37.738	10.202	0.592	0.049	0.859	0.026
		Average	39.375	9.627	0.588	0.048	0.873	0.024
GPR_Lassso	WI	y1	41.99	9.299	0.585	0.047	0.884	0.023
GPR_Lassso	WI	y2	37.958	10.251	0.592	0.048	0.858	0.026
		Average	39.974	9.775	0.589	0.048	0.871	0.025
GPR_Ridge	WI	y1	33.521	7.205	0.57	0.048	0.905	0.02
GPR_Ridge	WI	y2	33.701	8.817	0.594	0.049	0.888	0.02
		Average	33.611	8.011	0.582	0.049	0.897	0.020
UPDN_1	I	y1	133.047	7.442	0.695	0.024	0.373	0.055
UPDN_1	I	y2	99.26	11.279	0.711	0.021	0.402	0.05
		Average	116.154	9.361	0.703	0.023	0.388	0.053
UPDN_2	I	y1	95.62	7.786	0.669	0.025	0.409	0.053
UPDN_2	I	y2	75.986	11.879	0.691	0.019	0.389	0.051
		Average	85.803	9.833	0.680	0.022	0.399	0.052
UPDN_3	I	y1	97.618	6.255	0.687	0.023	0.389	0.064
UPDN_3	I	y2	72.15	9.406	0.698	0.017	0.397	0.051
		Average	84.884	7.831	0.693	0.020	0.393	0.058
UPDN_4	I	y1	102.29	7.787	0.679	0.02	0.346	0.061
UPDN_4	I	y2	74.66	12.035	0.7	0.014	0.356	0.057
		Average	88.475	9.911	0.690	0.017	0.351	0.059
MPDN_1	I	y1	111.247	23.885	0.662	0.044	0.766	0.04
MPDN_1	I	y2	102.691	23.825	0.668	0.044	0.758	0.043
		Average	106.969	23.855	0.665	0.044	0.762	0.042
MPDN_2	I	y1	98.646	21.658	0.653	0.046	0.782	0.041
MPDN_2	I	y2	90.899	21.828	0.643	0.046	0.771	0.04
		Average	94.773	21.743	0.648	0.046	0.777	0.041
MPDN_3	I	y1	96.984	21.958	0.66	0.046	0.766	0.042
MPDN_3	I	y2	89.359	22.391	0.638	0.047	0.769	0.041
		Average	93.172	22.175	0.649	0.047	0.768	0.042
MPDN_4	I	y1	94.62	21.06	0.651	0.046	0.779	0.039
MPDN_4	I	y2	85.397	21.374	0.632	0.047	0.78	0.038
		Average	90.009	21.217	0.642	0.047	0.780	0.039
GPR_L1_0.5	I	y1	64.279	14.495	0.624	0.046	0.825	0.031
GPR_L1_0.5	I	y2	48.417	10.777	0.623	0.048	0.783	0.04
		Average	56.348	12.636	0.624	0.047	0.804	0.036
GPR_L1_0.25	I	y1	62.199	13.912	0.61	0.046	0.83	0.03
GPR_L1_0.25	I	y2	46.086	10.418	0.621	0.048	0.787	0.04
		Average	54.143	12.165	0.616	0.047	0.809	0.035
GPR_L1_0.75	I	y1	65.515	14.917	0.627	0.045	0.824	0.032
GPR_L1_0.75	I	y2	49.455	11.066	0.624	0.048	0.781	0.039
		Average	57.485	12.992	0.626	0.047	0.803	0.036
GPR_Lassso	I	y1	67.005	15.7	0.628	0.046	0.82	0.033
GPR_Lassso	I	y2	50.073	11.395	0.624	0.048	0.78	0.04
		Average	58.539	13.548	0.626	0.047	0.800	0.037
GPR_Ridge	I	y1	116.056	22.719	0.788	0.049	0.857	0.028
GPR_Ridge	I	y2	86.785	18.019	0.775	0.049	0.826	0.032
		Average	101.421	20.369	0.782	0.049	0.842	0.030

### Prediction performance under data set 1

First, we compared the prediction performance of the 13 models across environments and traits without taking into account the genotype by environment interaction. Table 2 shows that the best predictions for data set 1 under MSE were under model GPR_Ridge with an MSE= 33.6, while the worst prediction was under model UPDN_1 with an MSE= 50.556. This means that the best models (GPR_Ridge) outperformed the worst model in terms of MSE by =((50.556-33.6)×100/33.6)= 50.464%. Under MAAPE, the best predictions were obtained under models GPR_Ridge and MPDN_1 with a MAAPE = 0.582, while the worst model was UPDN_3 with a MAAPE = 0.652. Thus, the GPR_Ridge and MPDN_1 models outperformed the UPDN_3 model by ((0.652-0.582) ×100/0.582)= 12.0274%. In terms of Pearson’s correlation, the best performance was also observed under model GPR_Ridge, with an APC = 0.897, while the worst performance was observed under model UPDN_1 with an APC = 0.5350, proving the superiority of GPR_Ridge over UPDN_1, which was equal to ((0.897-0.5350) ×100/0.5350)= 67.66355% (Table 2).

Now we present the results for data set 1 taking into account genotype by environment interaction. Under MSE, the GPR_L1_0.25 (MSE = 54.10) model was the best in terms of prediction performance, while the worst was UPDN_1 (MSE = 116.154). The superiority of the GPR_L1_0.25 over UPDN_1, was equal to ((116.154-54.10) ×100/54.10)= 114.7024%. In terms of MAAPE, the best model was also model GPR_L1_0.25 with a MAAPE= 0.615, but now the worst model was GPR_Ridge with a MAAPE= 0.782; for this reason, the GPR_L1_0.25 model outperformed the GPR_Ridge model by ((0.782-0.615) ×100/0.615)= 26.969%. Finally, in terms of APC, the best model was GPR_Ridge (APC = 0.841), while the worst was model UPDN_4 with APC = 0.351, which means that the best model (GPR_Ridge) outperformed the worst model (UPDN_4) in terms of APC by ((0.841-0.351) ×100/0.351)= 139.601% (Table 2).

**Figure 4:**
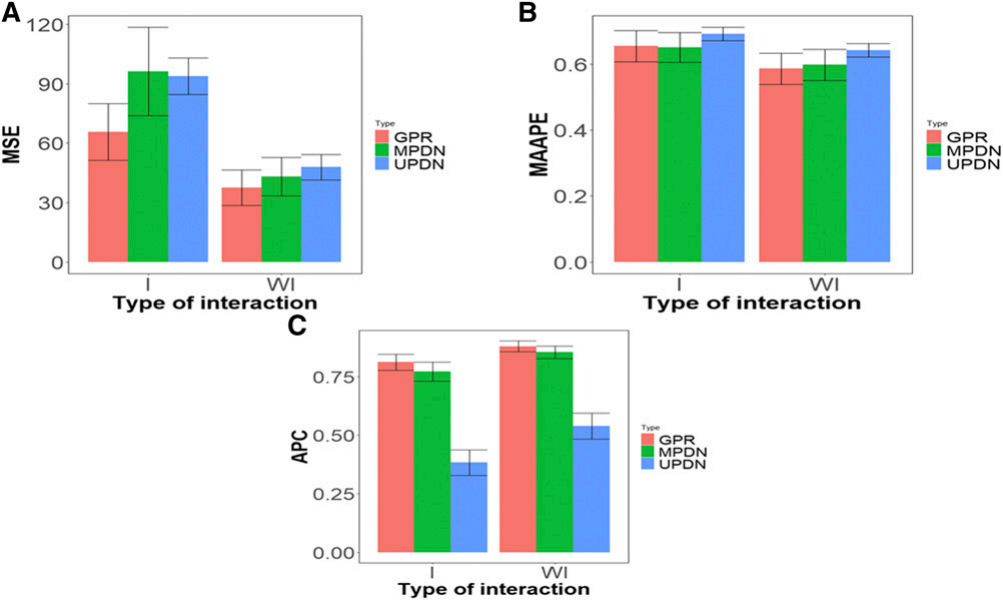
Prediction performance of data set 1 in terms of mean square error (MSE) (A), mean arctangent absolute percentage error (MAAPE) (B) and Average Pearson Correlation (APC) (C) across the traits and models of the same type. GPR denotes the univariate generalized Poisson regression model, MPDN denotes multivariate Poisson deep neural network and UPDN denotes univariate Poisson deep neural network.

Figure 4 provides a summary of the five generalized regression models (GPR_L1_0.75, GPR_L1_0.5, GPR_L1_0.25, GPR_Lasso, GPR_Ridge), the four multivariate Poisson deep learning models (MPDN_1, MPDN_2, MPDN_3, MPDN_4) and the four univariate Poisson deep learning models (UPDN_1, UPDN_2, UPDN_3, UPDN_4). Figure 4A shows that in terms of MSE, the best predictions were observed when the genotype by environment interaction was ignored. For example, under the generalized Poisson regression models (GPR), we can observe in Figure 4A, that without interaction in terms of MSE, they outperformed by ((65.6-37.5) ×100/37.5)= 74.933% those models with the interaction term, while under the MPDN models when the interaction term was ignored, the performance was better by ((96.2-43.1) ×100/43.1)= 123.202%. Also, under the UPDN, the prediction performance was better without the interaction term than when it was taken into account, by ((93.8-47.8) ×100/47.8)= 96.23%. This provides empirical evidence that for this data set, taking into account the genotype by environment interaction did not help improve the prediction performance. In general, we did not find statistical differences between the prediction performances of the univariate generalized Poisson regression and MPDNN models; however, when the genotype by environment interaction was taken into account, the GPR model outperformed the MPDN by ((96.2-65.61) ×100/65.61)= 46.631%, while when the interaction term was ignored, the GPR was better than the MPDN models by ((43.1-37.5) ×100/37.5)= 14.933%. It is very important to point out that without the interaction term, the best and worst performance was under the GPR models and the worst under the UPDN model. When the interaction term was taken into account, the GPR was also the best, but now the worst performance was observed under the MPDN (Figure 4A).

In terms of MAAPE, we found no statistical differences between taking into account genotype by environment interaction and ignoring it in the three models (GPR, MPDN and UPDN). But results showed a gain of ((0.654-0.586) ×100/0.586)= 11.604% under the GPR when the genotype by environment interaction was ignored, compared to when it was taken into account. However, this gain was of ((0.651-0.598) ×100/0.598)= 8.863% and ((0.691-0.642) ×100/0.642)= 7.632% under MPDN and UPDN, respectively. Also, in general in terms of MAAPE, between the three types of models, GPR, MPDN and UPDN, we found no statistical differences since the performance of the three models was very similar (Figure 4B); however, in general terms, the UPDN was the worst. In terms of Pearson’s correlation, we found the three models performed better when ignoring genotype by environment interaction. For example, the GPR that ignored genotype by environment interaction outperformed the models with the interaction term by ((0.879-0.811) ×100/0.811)= 8.385%, while the MPDN and UPDN outperformed the models with genotype by environment interaction by ((0.854-0.771) ×100/0.771)=10.765% and ((0.538-0.383) ×100/0.383)= 40.469%, respectively. However, in terms of Pearson’s correlation, we found no statistical differences in prediction performance between the GPR and MPDN models (Figure 4C). Also, under Pearson’s correlation, the worst performance was under the UPDN and the best under the GPR and MPDN models (Figure 4C).

### Prediction performance of data set 2

We compared the prediction performance of the 13 models across environments and traits, first without taking into account the genotype×environment (WI) interaction term and then taking into account the interaction term (I). First, under the MSE, the best model was GPR_L1_0.25 with a MSE= 8.56, while the worst was UPDN_1 with a MSE= 12.429, which means that GPR_L1_0.25 outperformed the UPDN_1 model by ((12.429-8.56)×100/8.56)= 45.198% (Table 3). Under the MAAPE, the best model was MPDN_4 with a MAAPE = 0.357, while the worst was UPDN_1 with a MAAPE = 0.420; for this reason, MPDN_4 outperformed UPDN_1 by ((0.420-0.357)×100/0.357)=17.647%. Under the APC, the best and worst models were MPDN_2 (APC= 0.556) and UPDN_1 (APC= 0.444), respectively, which means that the best model (MPDN_2) outperformed the worst model by ((0.556-0.444)×100/0.444)= 25.225%.

**Table 3:** Prediction performance of data set 2 in terms of mean square error (MSE), mean arctangent absolute percentage error (MAAPE) and Average Pearson Correlation (APC) without taking into consideration the genotype×environment interaction (WI) and taking it into account (I) in the 13 models. SE_1 denotes the standard error of the MSE, SE_2 denotes the standard error of the MAAPE and SE_3 denotes the standard error of the APC

Model	Interaction	Trait	MSE	SE_1	MAAPE	SE_2	APC	SE_3
UPDN_1	WI	PTR	16.682	3.268	0.503	0.025	0.371	0.045
UPDN_1	WI	SB	9.640	0.816	0.408	0.019	0.408	0.029
UPDN_1	WI	SN	10.965	1.144	0.350	0.016	0.552	0.037
		Average	12.429	1.743	0.420	0.020	0.444	0.037
UPDN_2	WI	PTR	11.323	1.172	0.438	0.019	0.458	0.039
UPDN_2	WI	SB	8.244	0.671	0.383	0.021	0.449	0.041
UPDN_2	WI	SN	8.786	0.716	0.306	0.014	0.622	0.032
		Average	9.451	0.853	0.376	0.018	0.510	0.037
UPDN_3	WI	PTR	12.444	1.38	0.462	0.023	0.458	0.043
UPDN_3	WI	SB	7.833	0.562	0.379	0.016	0.467	0.044
UPDN_3	WI	SN	8.542	0.621	0.313	0.014	0.634	0.033
		Average	9.606	0.854	0.385	0.018	0.520	0.040
UPDN_4	WI	PTR	10.651	0.863	0.421	0.022	0.492	0.032
UPDN_4	WI	SB	7.382	0.493	0.373	0.018	0.487	0.038
UPDN_4	WI	SN	8.364	0.835	0.294	0.015	0.64	0.034
		Average	8.799	0.730	0.363	0.018	0.540	0.035
MPDN_1	WI	SN	8.287	0.815	0.300	0.015	0.644	0.031
MPDN_1	WI	PTR	11.433	0.892	0.438	0.019	0.514	0.034
MPDN_1	WI	SB	8.060	0.683	0.380	0.016	0.474	0.038
		Average	9.260	0.797	0.373	0.017	0.544	0.034
MPDN_2	WI	SN	8.362	0.816	0.292	0.015	0.649	0.031
MPDN_2	WI	PTR	10.523	0.791	0.429	0.019	0.538	0.033
MPDN_2	WI	SB	7.357	0.572	0.369	0.016	0.482	0.037
		Average	8.747	0.726	0.363	0.017	0.556	0.034
MPDN_3	WI	SN	8.312	0.790	0.304	0.015	0.656	0.030
MPDN_3	WI	PTR	10.252	0.801	0.420	0.019	0.544	0.033
MPDN_3	WI	SB	7.616	0.564	0.375	0.016	0.460	0.038
		Average	8.727	0.718	0.366	0.017	0.553	0.034
MPDN_4	WI	SN	8.233	0.796	0.278	0.015	0.653	0.030
MPDN_4	WI	PTR	10.371	0.778	0.422	0.019	0.528	0.033
MPDN_4	WI	SB	7.855	0.593	0.372	0.016	0.449	0.038
		Average	8.820	0.722	0.357	0.017	0.543	0.034
GPR_L1_0.5	WI	SN	8.389	0.787	0.304	0.015	0.630	0.031
GPR_L1_0.5	WI	PTR	10.139	0.769	0.408	0.019	0.535	0.033
GPR_L1_0.5	WI	SB	7.168	0.555	0.371	0.016	0.482	0.037
		Average	8.565	0.704	0.361	0.017	0.549	0.034
GPR_L1_0.25	WI	SN	8.384	0.786	0.304	0.015	0.631	0.031
GPR_L1_0.25	WI	PTR	10.132	0.769	0.408	0.019	0.534	0.033
GPR_L1_0.25	WI	SB	7.163	0.556	0.371	0.016	0.483	0.037
		Average	8.560	0.704	0.361	0.017	0.549	0.034
GPR_L1_0.75	WI	SN	8.389	0.787	0.304	0.015	0.631	0.031
GPR_L1_0.75	WI	PTR	10.140	0.769	0.408	0.019	0.535	0.033
GPR_L1_0.75	WI	SB	7.170	0.555	0.371	0.016	0.482	0.037
		Average	8.566	0.704	0.361	0.017	0.549	0.034
GPR_Lassso	WI	SN	8.390	0.787	0.304	0.015	0.631	0.031
GPR_Lassso	WI	PTR	10.140	0.769	0.408	0.019	0.535	0.033
GPR_Lassso	WI	SB	7.171	0.556	0.371	0.016	0.482	0.037
		Average	8.567	0.704	0.361	0.017	0.549	0.034
GPR_Ridge	WI	SN	8.346	0.785	0.303	0.015	0.627	0.032
GPR_Ridge	WI	PTR	10.262	0.767	0.413	0.019	0.512	0.035
GPR_Ridge	WI	SB	7.159	0.552	0.372	0.016	0.479	0.038
		Average	8.589	0.701	0.363	0.017	0.539	0.035
UPDN_1	I	PTR	16.213	1.677	0.503	0.024	0.326	0.047
UPDN_1	I	SB	10.089	0.923	0.404	0.021	0.361	0.044
UPDN_1	I	SN	12.308	1.113	0.353	0.019	0.494	0.051
		Average	12.870	1.238	0.420	0.021	0.394	0.047
UPDN_2	I	PTR	13.014	1.308	0.45	0.018	0.391	0.046
UPDN_2	I	SB	9.448	0.766	0.382	0.016	0.396	0.046
UPDN_2	I	SN	10.923	1.016	0.322	0.014	0.562	0.038
		Average	11.128	1.030	0.385	0.016	0.450	0.043
UPDN_3	I	PTR	13.346	1.472	0.456	0.019	0.43	0.045
UPDN_3	I	SB	9.061	0.598	0.384	0.015	0.431	0.04
UPDN_3	I	SN	10.718	1.042	0.323	0.018	0.575	0.047
		Average	11.042	1.037	0.388	0.017	0.479	0.044
UPDN_4	I	PTR	12.421	1.364	0.434	0.021	0.441	0.046
UPDN_4	I	SB	8.028	0.62	0.372	0.016	0.451	0.039
UPDN_4	I	SN	9.317	0.913	0.309	0.019	0.62	0.035
		Average	9.922	0.966	0.372	0.019	0.504	0.040
MPDN_1	I	SN	11.878	1.120	0.338	0.014	0.535	0.035
MPDN_1	I	PTR	15.276	1.231	0.487	0.018	0.391	0.040
MPDN_1	I	SB	10.068	0.776	0.400	0.015	0.370	0.041
		Average	12.407	1.042	0.408	0.016	0.432	0.039
MPDN_2	I	SN	11.503	1.044	0.323	0.014	0.595	0.031
MPDN_2	I	PTR	12.745	1.017	0.440	0.018	0.473	0.037
MPDN_2	I	SB	9.341	0.718	0.379	0.015	0.427	0.038
		Average	11.196	0.926	0.381	0.016	0.498	0.035
MPDN_3	I	SN	9.942	0.896	0.310	0.015	0.585	0.033
MPDN_3	I	PTR	11.654	0.910	0.424	0.019	0.471	0.037
MPDN_3	I	SB	8.226	0.622	0.375	0.016	0.425	0.038
		Average	9.941	0.809	0.370	0.017	0.494	0.036
MPDN_4	I	SN	10.314	0.914	0.297	0.015	0.589	0.033
MPDN_4	I	PTR	12.018	0.933	0.418	0.019	0.462	0.036
MPDN_4	I	SB	8.245	0.616	0.373	0.016	0.423	0.039
		Average	10.192	0.821	0.363	0.017	0.491	0.036
GPR_L1_0.5	I	SN	10.649	0.884	0.365	0.014	0.527	0.036
GPR_L1_0.5	I	PTR	11.539	0.864	0.434	0.018	0.462	0.035
GPR_L1_0.5	I	SB	8.482	0.622	0.400	0.016	0.347	0.040
		Average	10.223	0.790	0.400	0.016	0.445	0.037
GPR_L1_0.25	I	SN	10.661	0.883	0.366	0.014	0.526	0.036
GPR_L1_0.25	I	PTR	11.542	0.866	0.435	0.018	0.462	0.035
GPR_L1_0.25	I	SB	8.488	0.623	0.400	0.016	0.347	0.040
		Average	10.230	0.791	0.400	0.016	0.445	0.037
GPR_L1_0.75	I	SN	10.654	0.886	0.365	0.014	0.528	0.036
GPR_L1_0.75	I	PTR	11.557	0.865	0.434	0.018	0.460	0.035
GPR_L1_0.75	I	SB	8.475	0.621	0.400	0.016	0.347	0.040
		Average	10.229	0.791	0.400	0.016	0.445	0.037
GPR_Lassso	I	SN	10.652	0.886	0.365	0.014	0.528	0.036
GPR_Lassso	I	PTR	11.553	0.865	0.434	0.018	0.460	0.035
GPR_Lassso	I	SB	8.490	0.622	0.401	0.016	0.347	0.040
		Average	10.232	0.791	0.400	0.016	0.445	0.037
GPR_Ridge	I	SN	11.722	0.962	0.395	0.013	0.601	0.033
GPR_Ridge	I	PTR	11.986	0.885	0.438	0.018	0.503	0.035
GPR_Ridge	I	SB	8.444	0.659	0.386	0.015	0.461	0.038
		Average	10.717	0.835	0.406	0.015	0.522	0.035

Now when the genotype×environment interaction was taken into account under MSE, the best and worst models were UPDN_4 (MSE = 9.922) and UPDN_1 (MSE = 12.87), respectively. This means that the best model outperformed the worst by ((12.87-9.922)×100/9.922)=29.711%. Under the MAAPE, the best model was MPDN_4 (MAAPE = 0.363), while the worst was UPDN_1 (MAAPE = 0.402), and the best model outperformed the worst model by ((0.402-0.363)×100/0.363)= 10.743%. In terms of APC, the best model GPR_Ridge (APC = 0.522) outperformed the worst model UPDN_1 (APC = 0.394) by ((0.522-0.394)×100/0.394)= 32.487%.

**Figure 5:**
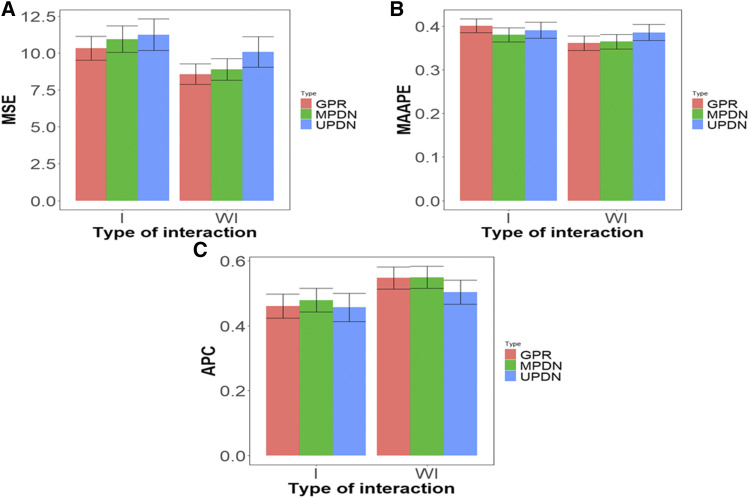
Prediction performance of data set 2 in terms of mean square error (MSE) (A), mean arctangent absolute percentage error (MAAPE) (B) and Average Pearson Correlation (APC) (C) across the traits and models of the same type. GPR denotes the univariate generalized Poisson regression model, MPDN denotes multivariate Poisson deep neural network and UPDN denotes univariate Poisson deep neural network.

Figure 5 summarizes the five generalized regression models (GPR_L1_0.75, GPR_L1_0.5, GPR_L1_0.25, GPR_Lasso, GPR_Ridge), the 4 multivariate Poisson deep neural networks (MPDN_1, MPDN_2, MPDN_3, MPDN_4) and the 4 univariate Poisson deep neural networks (UPDN_1, UPDN_2, UPDN_3, UPDN_4). In Figure 5A we can see that the generalized Poisson regression models (GPR) without interaction in terms of MSE statistically outperformed by ((10.3- 8.57) ×100/ 8.57)= 20.186% those models with the interaction term. However, under the MPDN models when the interaction term was ignored, the performance was statistically better by ((10.9- 8.89) ×100/ 8.89)= 22.609%, while the UPDN models, also under MSE, without the interaction term outperformed by ((11.2-10.1) ×100/10.1)= 10.891% those models with the interaction term. This provides empirical evidence that for data set 2, taking into account genotype by environment interaction did not help improve the prediction performance. Also, in this data set we found no statistical differences between the prediction performance of the generalized Poisson regression and MPDN and UPDN models; however, when the genotype by environment interaction was taken into account, the GPR model outperformed the MPDN and UPDN by ((10.9- 10.3) ×100/ 10.3)= 5.825% and ((11.2- 10.3) ×100/ 10.3)=8.737%, respectively; while when the interaction term was ignored, the GPR was better than the MPDN and UPDN models by ((8.89-8.57) ×100/ 8.57)= 3.734% and ((10.1-8.57) ×100/ 8.57)= 17.852%, respectively.

In terms of MAAPE, we only found statistical differences between taking into account the genotype by environment interaction and ignoring it in the GPR models; without genotype by environment interaction, they outperformed by ((0.401- 0.361) ×100/ 0.361)= 11.080% those models with genotype by environment interaction. Also, under MPDN and UPDN models without the interaction term outperformed by ((0.380- 0.365) ×100/ 0.365)= 4.109% and ((0.391- 0.386) ×100/ 0.386)=1.29%, respectively, those models with the interaction term. However, no statistical differences were observed between GPR and MPDN and UPDN models in both scenarios with and without genotype by environment interaction (Figure 5B), but in general the worst prediction performance was observed under the UPDN model. Finally, in terms of Pearson’s correlation under the GPR models, we also found statistical differences between models taking into account genotype by environment interaction and models ignoring it; those models without genotype by environment interaction outperformed by ((0.547- 0.460) ×100/ 0.460)= 18.913% (under GPR), by ((0.549- 0.479) ×100/ 0.479)= 14.613% (MPDN) and by ((0.503- 0.456) ×100/ 0.456)= 10.307% (under UPDN) those models with the interaction term. However, no statistical differences were found between the three models (GPR, MPDN and UPDN) in terms of APC with and without the interaction term (Figure 5C).

## Discussion

Due to the lack of multi-trait prediction models for count data, in this study we propose a multi-trait deep neural network for count data. Our proposed model is useful for the following reasons: (a) it is a multi-trait approach for count data, (b) it is powerful enough to capture linear and nonlinear patterns since it was built under the umbrella of deep learning models, (c) it is powerful when used with moderate or large data sets since the training process is performed using batches of the whole training set, thus avoiding memory problems; its implementation is possible using Tensorflow as back-end and Keras as front-end, and (d) it works with raw inputs like images, or other non pre-preprocessed inputs since this model is under the umbrella of deep neural networks (Pound *et al.*, 2017). For these reasons, since the proposed MPDN can be implemented in Keras, it is a very friendly and powerful framework for moderate or large data sets. Also, the ability of the proposed MPDN to capture nonlinear patterns is due to the fact that it belongs to models called “artificial deep neural networks” that are inspired in the biological functioning of the brain, and that work by stacking many layers with hundreds or thousands of neurons in each layer. The larger the number of stacked layers, the more powerful the model for capturing non-linear patterns due to the fact that in each layer, a specific non-linear transformation is applied to its inputs (Haykin 2009). The power of the proposed MPDN is theoretically supported by the approximation theorem that states that under artificial deep neural networks we can approximate any function to the desired degree with a large enough number of neurons (Cybenko 1989; Hornik 1991). However, it is important to point out that Keras/Tensorflow is a very flexible framework for implementing deep neural networks since without a strong background in mathematics and computer science, the user can implement univariate models for continuous, binary, categorical, count data, and multivariate models with any type of response variable including mixed response variables (Chollet and Allaire 2017).

The proposed MPDN model also has its disadvantages; some of them are: (a) we need to tune many hyper-parameters, which is still a very time-consuming process in all deep neural networks models, since there are no well-established methods and most of them are more art than science, (b) in the proposed MPDN, a covariance (correlation) matrix is not estimated to more efficiently capture the degree of similarity between traits; for this reason, many times multivariate deep learning models do not outperform univariate deep neural networks since the clear advantage of multivariate deep neural networks over the univariate deep neural network model is the amount of data used for training the model, and (c) there is a lot of empirical evidence that, in general, deep neural networks outperform conventional statistical machine learning methods when the number of observations used to train the model is very large, and the larger the better (Patterson and Gibson 2017), which is not easy in the context of genomic selection since most of the time in data sets collected in the field, there are few observations and a very large number of independent variables (markers). Also, the proposed MPDN cannot be used to estimate breeding values since breeding values, as pointed out by one reviewer, are additive effects and the proposed MPDN incorporates non-additive effects (Varona *et al.*, 2018). Also, the proposed MPDN is not able to decompose the genetic variance orthogonally into additive, dominance, additive×additive, dominance×additive, etc., variance components, since this orthogonal decomposition is valid only under restricted assumptions such as linkage equilibrium, random mating and no inbreeding (Gianola *et al.*, 2006). Despite these limitations, the proposed MPDN is attractive and fills the lack of multi-trait models for count data that can be implemented for moderate and large data sets. Also, its implementation is very friendly since it can be implemented using Keras as front-end and Tensorflow as back-end.

Another important matter that needs to be taken into account in the implementation of deep neural networks is the choice of the network architecture (topology). In this application, we used a fully connected network (feedforward network), where the information always flows in one direction. However, there are other topology options like the convolutional neural networks that are very efficient for images as inputs that are the state of the art for deep learning applications where the inputs are raw images; these topologies are very efficient for capturing correlated patterns in the inputs. Also, using this topology there are successful applications in the context of genomic selection like those of Ma *et al.* (2018) and Waldmann *et al.* (2020) that in these particular applications outperformed feedforward networks. Other deep neural network topologies are recurrent neural networks that are more appropriate for time series data because they allow previous outputs to be used as inputs (Chollet and Allaire 2017).

We found that, in terms of prediction performance, the MPDN outperformed its univariate Poisson deep neural network counterpart in both data sets, which can be attributed in part to the fact that for the training process it used more data, which makes the training process more efficient in capturing better complex patterns in in the data. However, our results also provide evidence that, in terms of prediction performance, the proposed multi-trait Poisson deep neural network model does not outperform the conventional univariate generalized Poisson regression, since in most cases, we found no statistical differences between the GPR and MPDN models. This can be attributed in part to the fact that the data may not have strong nonlinear patterns and thus it is enough with linear models like generalized Poisson regression models, and also that our data sets are small with regard to the number of observations. However, although the MPDN was not better in terms of prediction performance than the GPR models, its performance is competitive and has the advantage that it can be implemented for moderate-to-large data sets and is able to capture nonlinear patterns in the data when they are present. For these reasons, the proposed MPDN model is an attractive tool for breeders for performing genomic selection with count multi-traits, and it can enrich the analytical tools available for genomic prediction for multi-trait count data with complex nonlinearities.

However, as one of the reviewers pointed out, a good model not only has good generalization performance, but it gives some insight into the workings of the system. For this reason, the current state-of-the-art of deep learning models are not really useful for inference and association studies, since their parameters (weights) many times cannot be interpreted as in many statistical models; also, since neither feature selection nor feature importance are obvious, for this reason, the DL methodology inhibits testing hypotheses about the biological meaning with parameter estimates. But there are nowadays current research in this direction for implementing appropriate DL models, that in addition to doing good prediction performance, allow an understanding of the biological significance of the outputs. This research is of paramount importance since there are still large difficulties in understanding the biological background and genetic architecture of many traits. Particularly for traits that are difficult or expensive to measure in (poorly defined) phenotypes, where the relationship between genome and phenome is far from being understood.

It is important to point out that the proposed MPDN model is very flexible since it allows using raw inputs as images and other non-preprocessed inputs that cannot be applied directly with most conventional statistical machine learning methods used in genomic selection. For these reasons, the proposed MPDN can be used in other domains like biomedical informatics (Du *et al.*, 2011) finance, health science, marketing, etc., where there is a great need for predicting multivariate counts as a function of complex inputs.

Finally, although the proposed model was evaluated with only two data sets, the results provide evidence that it is competitive with univariate deep learning tools and conventional statistical learning tools and has the advantage that it can capture nonlinear patterns better than generalized Poisson regression and can be implemented in existing software (such as Keras as front-end and Tensorflow as back-end) that is very user friendly. Also, like all deep neural networks, the proposed MPDN will perform better than conventional statistical learning models in the context of large data sets, complex input information like images and very complex nonlinear patterns. However, more applications are needed in the context of genomic selection to gain more insight into the power of these models.

## Conclusions

A model for count multi-trait data were proposed under a multivariate deep neural framework. The proposed MPDN model can be implemented using Tensorflow as back-end and Keras as front-end, and for this reason, it can be implemented for moderate and large data sets in a very user friendly environment. Also, due to the fact that the proposed MPDN model is an artificial deep neural network model, it is able to capture nonlinear patterns by including in the specification of the network more than one hidden layer, which applies nonlinear transformation to the data to be able to capture these complex patterns. We found that the proposed MPDN outperformed the univariate Poisson deep neural network model, but it was not better than the generalized univariate Poisson regression models using two real data sets; thus more research is needed to prove the power of the MPDN model in the context of genomic selection. Although we obtained evidence that the proposed MPDN is competitive with regard to univariate deep learning models and conventional generalized Poisson regression models and is able to fill the lack of multi-trait predictions for count data in genomic selection, it also allows using raw inputs like images (which is not straightforward in conventional genomic prediction models) and its implementation does not require a lot of knowledge of statistics, machine learning and computer science since the libraries that currently exist for implementing these models are very user friendly.
